# Cases of Affection of Upper Jaw

**Published:** 1848-10

**Authors:** 


Cases of Affection of Upper Jaw.—At a recent meeting of the *Pathological Society of London,* the following cases were related by M. Fergusson:

*Osseous Tumor Removed from the Face of a Girl, aged fourteen, involving the Right Superior Maxilla and the Malar Bone.*

Enlargement was noticed six or seven years before the operation, and seemingly commenced in the alveolar ridge. There was no trace of the antrum. Patient made a favorable recovery, and has remained well since.

*Osteo-sarcomatous Tumor from the face of a Woman, aged thirty-six, involving the greater part of the Left Superior Maxilla.*

The tumor projected slightly into the mouth, and filled the interior of the antrum. Portions of the alveoli had been removed on two occasions previous to the last operation, when the whole mass, including the malar bone, was extirpated. The operation was performed about three months ago, and there has been no indication of return of the disease. The reappearance of the disease, which does not indicate malignancy in its physical characters, may probably admit of explanation, by reference to the manner in which the growth has extended in an upward direction, so that it was not isolated by the horizontal sections of the alveoli which had been made for its removal at the first operation.

*Portion of a Medullary Tumor occupying the site of the Superior Maxilla, removed after death, from a man, aged fifty-four.*

An operation had been proposed at an early period; but the patient refused to submit. The tumor grew rapidly, and he was anxious for an operation when it was too late. It extended to the base of the cranium, and had all the characters of malignancy.

Dr. Bentley exhibited a tumor of the lower jaw, which had been removed by Mr. Key. The patient, a boy, aged thirteen, was admitted into Guy's Hospital in October, 1841. He was pale and sallow, with a high and somewhat projecting forehead, light hair and eyes, and emaciated appearance. In consequence of his not being able to articulate distinctly, some difficulty was experienced in obtaining a history of the case. The following particulars were noted at the time:

About two years ago, a small lump appeared on the anterior part of the lower jaw, gradually increasing and extending backwards, forcing out, after a time, many of the lower teeth. Has never experienced any pain. The jaw gradually increased in size, when he applied to a surgeon, who tried various remedies without relief. Has always enjoyed a fair share of health, and can eat and drink well.

When admitted into the hospital, the lower jaw was apparently much enlarged and thickened, and advanced beyond the upper, having resting upon the upper part of the symphisis a globular, vascular sarcoma, which about fourteen days back, had begun to ulcerate on the surface, and at intervals bled profusely. Styptics were applied to arrest hemorrhage.

Seven days after admission, Mr. Key determined to remove the diseased part, which was accomplished in the following manner:—An incision was made along either side of the horizontal ramus of lower jaw, commencing from the angle in the hollow of the neck, meeting each other in the mesial line; the flap was turned up, and the cut vessels immediately secured; the lower jaw was sawn through, just anterior to its angles, detaching a small portion of the masseter muscle on each side. The jaw was carefully dissected out, the flap brought together, and sutures applied.

On the fourth day, the sutures were removed, and the whole of the wound had nearly healed. He left the hospital on the 9th of November, having had no bad symptom, and is now in perfect health.

The condition of the jaw prior to and after the operation was illustrated by drawings.

Dr. Hall Davis exhibited *Microscopical Preparations of Mollities Ossium*, with which a woman, dying from rare uterine disease, the particulars of which had been submitted to the consideration of the Society some weeks back, had been affected. Dr. Davis had previously submitted some specimens to the examination of Dr. Sharpey, Mr. Dalrymple, and Mr. John Quekett, who had remarked that the Haversian canals were much larger than natural; as also the bone cells; and in parts, near the edges of the bone, forming the wall of the Haversian canals; the canaliculi were but slightly visible, or had entirely disappeared. The bone along the edges above mentioned was extremely transparent; and numerous nucleated cells, blood discs, and fatty matter, occupied the cancellated structure, and some of the Haversian canals. The bone had also been examined chemically by Dr. Garrod, who found that 100 parts dried at 212° yielded—

| | |
|---|---|
| Phosphate of lime, | 16.40 |
| Carbonate of lime, with phosphate of magnesia, | 4 88 |
| Fatty matter, | 20 35 |
| Gelatine yielding matter, | 58.37 |
| | 100.00 |

thus exhibiting a considerable difference from the proportions given for healthy bone by Berzelius—viz.

| | |
|---|---|
| Earthy matters, | 66.70 |
| Animal matters, | 33.30 |

The history of the case was then detailed, and it appeared that the female had, at the commencement of the affection, lived in a dry climate, and for the last six years of her life in Lancashire, on the borders of a lake surrounded by woodland. There had been no hereditary tendency, the father and mother being still alive, as also five other children, all enjoying perfect health. She had never been affected with rheumatism, and died from an acute attack of pneumonia.

